# Olive baboons' (*Papio anubis*) response towards crowned eagles (*Stephanoaetus coronatus*) at Lake Manyara National Park

**DOI:** 10.5194/pb-4-101-2017

**Published:** 2017-05-15

**Authors:** Filipa M. D. Paciência, Deusdedith Baluya, Pay Mbaryo, Sascha Knauf, Dietmar Zinner

**Affiliations:** 1 Cognitive Ethology Laboratory, German Primate Center, Leibniz Institute for Primate Research, Kellnerweg 4, 37077 Göttingen, Germany; 2 Tanzania National Parks, P.O. Box 3134, Arusha, Tanzania; 3 Work Group Neglected Tropical Diseases, Pathology Unit, German Primate Center, Leibniz Institute for Primate Research, Kellnerweg 4, 37077 Göttingen, Germany

## Abstract

In this paper we report on two encounters between olive baboons (*Papio anubis*) and crowned eagles (*Stephanoaetus coronatus*) at Lake Manyara National Park, northern Tanzania. During these encounters olive baboons responded by giving alarm calls and all infants and juveniles rushed down from trees seeking cover under bushes or close proximity to adult conspecifics. In one of the events, alarm calls from banded mongoose (*Mungos mungo*) and rock hyraxes (*Procavia capensis*) most likely triggered alarm calling of vervet monkeys (*Chlorocebus pygerythrus*) which in turn prompted baboons to respond with alarm calls as well. In both observations, adult male baboons took the lead in climbing trees, threatening the eagle (staring, yawning, ground slapping) and chasing it away. The reaction of the baboons suggests that crowned eagles pose a threat at least for juvenile baboons at Lake Manyara National Park.

## 1 Introduction

**Figure 1:**
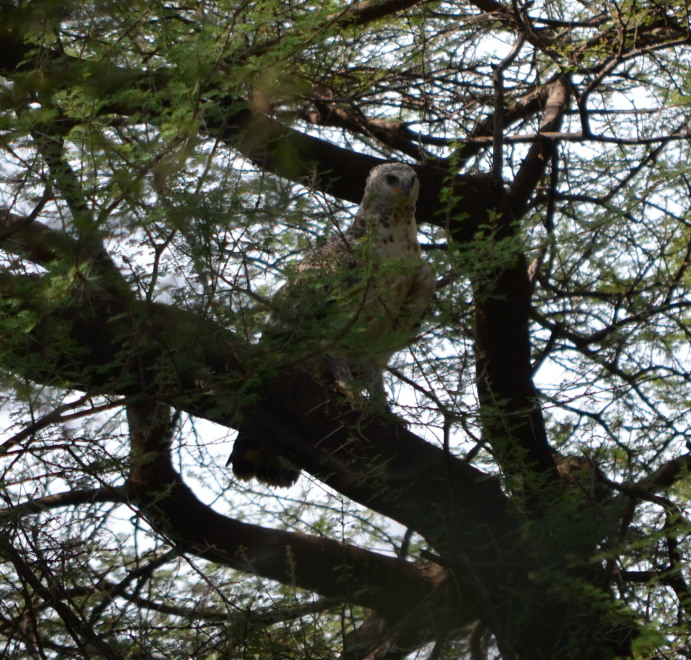
Immature crowned eagle (*Stephanoaetus coronatus*) near Endala Research Station, Lake Manyara National Park.

Predation is seen as a powerful selective force that has shaped the behaviour of primates in many respects (van Schaik, 1983; Terborgh and Janson, 1986; Janson, 1992; Isbell, 1994) and raptors are known to be common predators of primates (Hart, 2000; Miller and Treves, 2011; McGraw and Berger, 2013). In Africa, one of the most reported aerial predators of primates is the crowned eagle *Stephanoaetus coronatus* (Fig. 1; Skorupa, 1989; Struhsaker and Leakey, 1990; Leland and Struhsaker, 1993; Maisels et al., 1993; Hart et al., 1996; Zuberbühler, 2000, 2001; Mitani et al., 2001; Shultz, 2001; Sanders et al., 2003; Rainey et al., 2004; Arnold et al., 2008; McPherson et al., 2015; Malan et al., 2016). This eagle is widely distributed in sub-Saharan Africa with a preference for forests and woodlands (Skorupa, 1989; Ferguson-Lees and Christie, 2001). Crowned eagles have a body mass of 3.4 to 4.1 kg and a length from 81 to 92 cm with females being larger than males. Their main targets are usually small- to medium-sized mammals, but they can also kill animals heavier than themselves such as bushbucks *Tragelaphus scriptus* (30 kg; Brown, 1971, 1982; Daneel, 1979; Steyn, 1973, 1983; Tarboton, 1989). Due to the eagle's habitat and prey preferences, arboreal primates are among its most frequent targets (Isbell, 1994; Shultz, 2002). More terrestrial primates such as baboons, mandrills (*Mandrillus sphinx*) and mangabeys (*Cercocebus atys*) have also been found among their prey (Jouventin, 1975; Mitani et al., 2001; Sanders et al., 2003; Shultz et al., 2004; McGraw et al., 2006).

In this paper we report on the interaction between an olive baboon group and crowned eagles at Lake Manyara National Park (LMNP). Although, as in many other parts of their range, the main predators of olive baboons are most likely lions (*Panthera leo*) and leopards (*Panthera pardus*; Cowlishaw, 1994; Palombit, 2013), large eagles may pose an additional threat to immature baboons. If this is the case, we expect to observe anti-predator behaviours of baboons including alarm calls, mobbing or even counter-attack. Here we provide information on baboon behaviour when facing a potential aerial predator, thus contributing to the scarce data on baboon–raptor interaction.

## 2 Methods

**Figure 2:**
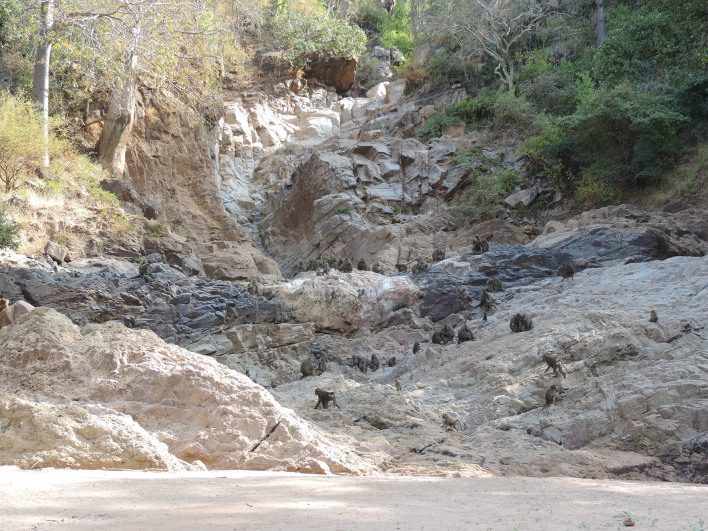
Members of the baboon study group at the cliff in Endala Research Station, Lake Manyara National Park.

The observations took place on 15 June and 21 September in 2016 at LMNP (approx. 3${}^{\circ }$28.616${}^{\prime }$ S 35${}^{\circ }$46.757${}^{\prime }$ E) in northern Tanzania. LMNP is home to five primate species: the lesser bushbaby (*Galago senegalensis*), the large-eared greater galago (*Otolemur crassicaudatus*), the vervet monkey (*Chlorocebus pygerythrus*), the blue monkey (*Cercopithecus mitis*) and the olive baboon (*Papio anubis*). The events described below occurred near the Endala Research Camp, which lies at the base of the Great Rift Valley. The habitat mainly comprises bush and Acacia woodland, with large trees along the riversides. Endala has a permanent water source, which attracts many wildlife species including baboons, especially during the dry season. Together with its proximity to the steep rocky escarpment, Endala seems to be one of the preferred sleeping sites of many baboon groups in the area (Fig. 2). Olive baboon groups at LMNP are usually larger than in other parts of their range (personal observation). Our focal group is the largest studied to date, with more than 170 individuals (including at least 35 adult males and 53 adult females). The group was habituated to human observers on foot in 2015. Since then, it has been part of a research project for which we gathered data, particularly on the behaviour of oestrous females. The incidents reported here happened during data collection while following the baboons at a distance of less than 10 m. The eagle encounters were directly observed and recorded on site with a voice recorder and later transcribed to a research diary.

Beside crowned eagles, several other large raptors occur in LMNP. Although immature crowned and martial eagles (*Polemaetus bellicosus*) are easily mistaken for each other, the eagles in our observations were clearly identified as crowned eagles due to their dark spotted legs.

### Ethics statement

This study was carried out in accordance with the Tanzania Wildlife Research Institute's Guidelines for Conducting Wildlife Research and with permission from Tanzania National Parks (TNP/HQ/C.10/13). Additional research permission was granted by the Commission for Science and Technology in Tanzania (2016-115-NA-2014-228).

## 3 Results

We witnessed two close encounters between members of the habituated olive baboon group and a crowned eagle. In both occasions there was a clear sky and good visibility.

### 3.1 Observation 1, 15 June 2016, around 10:10 h

The baboon group was scattered between Acacia trees (*Acacia tortilis*). At the time of the event around 25 baboons (males and females) could be observed foraging or resting. Suddenly some adult individuals gave alarm calls and stared in a certain direction. We followed their gaze and spotted a crowned eagle (Fig. 1) perched in one Acacia tree near the group (15 m away from the group centre). The baboon infants immediately ran towards their mothers while moaning (Maciej, et al., 2013) and the juveniles rushed down from the trees into bush cover while also giving alarm calls. One adult male ran to the tree where the eagle was and climbed it quickly while threat grunting. In the tree in front of the raptor, three other adult males were staring and yawning (threatening) at the bird. The quick approach from the first male made the eagle fly to the next tree (ca. 5 m). The sequence recurred several times, the same male climbed the tree where the eagle was and the others moved through the branches in the neighbouring trees while threatening the eagle (staring, yawning and hand slap on the tree). They all gave roar grunts from time to time. The eagle moved from tree to tree increasing the distance to the group to at least 100 m. This took less than 10 min. All females with young offspring remained vigilant and all juveniles stayed close to adult individuals until the eagle moved away. When the eagle crossed a dry river (more than 150 m away) the group relaxed and resumed their former activities.

### 3.2 Observation 2, 21 September 2016, around 17:30 h

**Figure 3:**
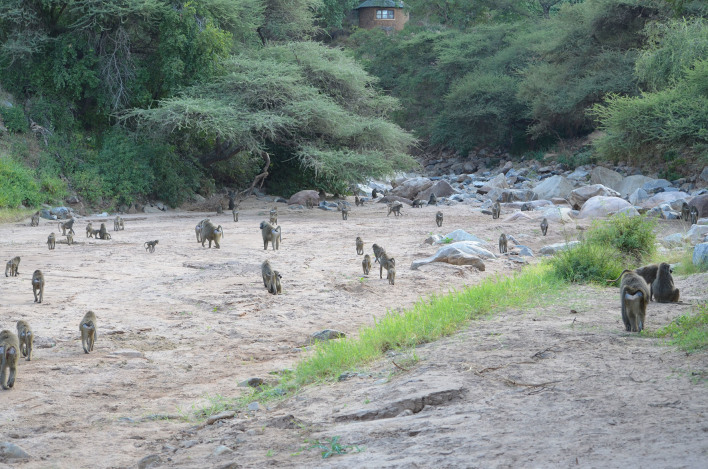
Members of the baboon study group crossing the dry river bed towards the cliff at Endala Research Station, Lake Manyara National Park. The second event took place around here (in the background a hut belonging to the research station can be seen).

At the time of the observation the majority of the group was resting in the rocky formations that shape Endala waterfall (Fig. 2). This is an open area with good visibility where at least 80 baboons could be seen mainly engaged in grooming or resting. After a while, several alarm calls were given by a group of banded mongoose (*Mungos mungo*) followed by more alarm calls from rock hyraxes (*Procavia capensis*). Both hyraxes and mongooses were located between the cause of the alarm and the baboon group (around 25 m away from each point). The baboons were all looking in the direction of the calls and some individuals even stood bipedally while surveying the area. After the hyrax calls, vervet monkeys joined as well giving alarm calls followed then by alarm calls from the baboons. At this time, many infant baboons ran toward their mothers or adult males and juveniles stopped playing while moving near to adults or sub-adults (0.5 m). Most of the baboons were looking in the same direction and some adult males changed their position and moved to the front of the group into the direction of the calls. One of them walked for 50 m in the direction of the suspected cause of the alarm and stared at the Acacia trees in the riverbed (Fig. 3). The mongooses, hyraxes and vervet monkeys continued alarm calling and remained behind. We followed the male baboon (leaving a distance of 20 m) and spotted a crowned eagle perched in one Acacia tree facing the male baboon's direction. The baboon gave an alarm call which triggered the group behind to alarm call as well. The male chased the perched eagle away by running with its mouth open in the direction of the tree. Threat grunting could be heard while the baboon was running. The eagle flew away, from tree to tree, as in the previous observation, while the baboon continued chasing it while yawning and grunting in between. When the bird flew more than 100 m away the baboon returned and sat half way between the eagle and the group while observing the movements of the bird. The rest of the group resumed its previous activities and eventually the eagle abandoned the place. Again, this observation took less than 10 min.

## 4 Discussion

Crowned eagles are probably the most effective aerial predators of primates in Africa (Mitani et al., 2001; Shultz, 2001; Shultz and Noë, 2002). In Taï forest 49 to 58 % of crowned eagles' prey were primates (Shultz, 2002; McGraw et al., 2006), whereas at other forest sites the numbers might even rise to more than 80 % (Kibale, Uganda, 80 %; Skorupa, 1989; Struhsaker and Leakey, 1990; Mitani et al., 2001; Kiwengoma Forest Reserve, Tanzania, 90 %; Msuya, 1993; Ituri Forest, Democratic Republic of Congo, 88 % Hart et al., 1996). Depending on habitat and abundance of prey species, arboreal primate taxa, such as guenons and colobines, constitute the major prey (Brown, 1971; Struhsaker and Leakey, 1990; Mitani et al., 2001). Reports of predation by crowned eagles on baboons are rare; though some studies have found remains of chacma baboons (*Papio ursinus*) in crowned eagle nests in South Africa (Jarvis et al., 1980; Boshoff et al., 1994). Also in Uganda (Ngogo and Kibale) young olive baboons were reported to be part of the diet of crowned eagles (Mitani et al., 2001; Sanders et al., 2003). Immature olive baboons with a body mass less than 10 kg fall well into the range of crowned eagles' prey.

Crowned eagles use stealth and surprise when hunting and employ different techniques such as sitting and waiting in the canopies or swooping down from a higher perch before striking (Brown, 1976; Gautier-Hion and Tutin, 1988; Cordeiro, 1992; Maisels et al., 1993). They are well adapted to hunt in forested habitats because they have, in contrast to eagles of open habitats, relatively short and round wings and a long tail, making fast flight manoeuvres in dense forest possible (Tarboton, 1989). LMNP comprises two large forest areas: a groundwater forest in the north and the Marang Forest in the south. These forests are known to hold crowned eagles (Cordeiro, 1992) and immature eagles may also visit other parts of the NP in search for an empty territory (Oatley et al., 1998). The eagle which we observed on the first event was most likely an immature eagle, found in a more open habitat.

When faced with potential threats, primates use different strategies to avoid aerial predation. Among them are the alarm calls, group congregation, canopy descent, hiding under cover and aggressive defence or counter-attack. Mobbing is common in many primate species (Hart, 2000). In groups with several males, they may team up to collectively threaten and attack a predator (Cordeiro, 1992; Maisels et al., 1993; Cowlishaw, 1994; Korstjens, 2001; Bettridge and Dunbar, 2013). Because of the sexual dimorphism in body and canine size in many primate species, including olive baboons (Swedell, 2011), males probably face a lower risk when mobbing, threatening and attacking predators and may be especially motivated to defend their reproductive resources (females) and their infants (Isbell, 1994). This is in accordance with our observations, where males chased the eagle away while nearby females remained vigilant with their offspring nearby. In other studies, crowned eagles were observed to trigger alarm calls in vervet monkeys, making all group members look up and to run into cover under bushes (Seyfarth and Cheney, 1990; Cheney and Seyfarth, 1996). We saw similar reactions by juvenile baboons during the first eagle event, which were also reported from baboons attacked by Verreaux's eagles, *Aquila verreauxii* (Hall, 1963; Boshoff et al., 1991; Cowlishaw, 1994; Zinner and Peláez, 1999).

During the second observation, banded mongoose, rock hyrax and vervet monkeys responded with alarm calls and flight behaviour after detecting the crowned eagle. Since mongooses and hyraxes are also reported as part of crowned eagles' diet (Jarvis et al., 1980; Boshoff et al., 1994; Swatridge et al., 2014), alarm reactions (calls) from these species are expected. Interspecific responses to alarm calls have been observed from different taxa. For primates it was reported that they distinguish and act accordingly when an alarm call is given by other primate species, mammals or birds (Seyfarth et al., 1980; Seyfarth and Cheney, 1990; Zuberbühler et al., 1997; Zuberbühler, 2000; Shultz, 2001). The recognition of alarm vocalizations given by distinct species in a predation context and an appropriate action is of adaptive value since it might increase the survival chances of the receivers.

## 5 Conclusions

African crowned eagles are widely recognized as the most important aerial predator for primates throughout sub-Saharan Africa. In LMNP, crowned eagles were observed twice near a group of olive baboons. The behaviour of the baboons suggests that crowned eagles are potential predators, at least for immature individuals. Group members all participated in vigilance but adult males were the ones which took the lead in chasing the eagle away in both incidents. Our observation of the second incident suggests that baboons responded to the alarm calls of vervet monkeys, hyraxes and mongooses. Reactions of primates to alarm calls by other species have been shown in previous studies (Noë and Bshary, 1997; Zuberbühler, 2000; Eckardt and Zuberbühler 2004). The sequence of alarm vocalizations by different species during the second event might suggest a cascade effect in the perception of alarm calls. However, we cannot exclude the theory that each species independently detected the eagle and reacted accordingly.

## Data Availability

No data sets were used in this article.
